# Reprogramming Malignant Cancer Cells toward a Benign Phenotype following Exposure to Human Embryonic Stem Cell Microenvironment

**DOI:** 10.1371/journal.pone.0169899

**Published:** 2017-01-09

**Authors:** Shufeng Zhou, Mohamed Abdouh, Vincenzo Arena, Manuel Arena, Goffredo Orazio Arena

**Affiliations:** 1 Cancer Research Program, McGill University Health Centre-Research Institute, Montreal, Canada; 2 Department of Experimental Surgery, Montreal General Hospital, McGill University, Montreal, Canada; 3 Deparment of Obstetrics and Gynecology, Santo Bambino Hospital, Catania, Italy; 4 Department of Surgical Sciences, Organ Transplantation and Advances Technologies, University of Catania, Catania, Italy; 5 Department of Surgery, St. Mary Hospital, McGill University, Montreal, Canada; University of Pécs Medical School, HUNGARY

## Abstract

The embryonic microenvironment is well known to be non-permissive for tumor development because early developmental signals naturally suppress the expression of proto-oncogenes. In an analogous manner, mimicking an early embryonic environment during embryonic stem cell culture has been shown to suppress oncogenic phenotypes of cancer cells. Exosomes derived from human embryonic stem cells harbor substances that mirror the content of the cells of origin and have been reported to reprogram hematopoietic stem/progenitor cells via horizontal transfer of mRNA and proteins. However, the possibility that these embryonic stem cells-derived exosomes might be the main effectors of the anti-tumor effect mediated by the embryonic stem cells has not been explored yet. The present study aims to investigate whether exosomes derived from human embryonic stem cells can reprogram malignant cancer cells to a benign stage and reduce their tumorigenicity. We show that the embryonic stem cell-conditioned medium contains factors that inhibit cancer cell growth and tumorigenicity *in vitro* and *in vivo*. Moreover, we demonstrate that exosomes derived from human embryonic stem cells display anti-proliferation and pro-apoptotic effects, and decrease tumor size in a xenograft model. These exosomes are also able to transfer their cargo into target cancer cells, inducing a dose-dependent increase in SOX2, OCT4 and Nanog proteins, leading to a dose-dependent decrease of cancer cell growth and tumorigenicity. This study shows for the first time that human embryonic stem cell-derived exosomes play an important role in the tumor suppressive activity displayed by human embryonic stem cells.

## Introduction

The embryonic microenvironment is well known to be nonpermissive for tumor development and possesses the unique ability to reprogram and reverse tumorigenicity [1–3]. Pioneer work demonstrated that carcinoma cells are reprogrammed when injected into a mouse blastocyst resulting in normal tissue originating from tumour cells in chimeric mice [4, 5]. It was also reported that the tumorigenicity of melanoma cells is reduced when they were implanted in vivo in embryos [6–8]. Moreover, adult human stem cells have also been shown to display inhibitory effects on cancer cells growth [9, 10].

Human embryonic stem cells (hESCs) are pluripotent stem cells derived from the inner cell mass of blastocysts and can be maintained virtually indefinitely undifferentiated in culture [11, 12]. They have an unlimited potential to proliferate in an undifferentiated state and have the ability to differentiate into most cell types [13, 14]. It has been reported that hESCs microenvironment can reprogram malignant cancer cells toward a less malignant and more differentiated cell phenotype [1, 15, 16]. The proposed mechanism by which hESCs microenvironment reprograms malignant cancer cells was related to Nodal related pathway [15]. More recently, Lefty proteins encased in exosomes derived from hESC have been shown to be involved in such process [17]. The fact that the reversal of the tumorigenicity of cancer cells observed in these studies was only partial, suggest that cooperation of other hESCs factors may be required to fully inhibit the expression of the malignant phenotype. Melanoma cells cultured on hESCs-conditioned matrigel have been shown to lose their invasive potential and to re-express specific melanocyte markers. These effects were not seen when these cells were cultured in hESCs-conditioned medium (hESCs-CM) [15, 18, 19]. In contrast, other studies reported that the exposure of cancer cells to hESCs-CM inhibited their tumorigenicity due to reduced proliferation and increased apoptosis [20]. In the present study we sought to determine the origin of the factors involved in these antitumorigenic effects.

Exosomes are small (30–100 nm) extracellular membrane-enclosed vesicles that originate from cellular endosomal compartment under both physiological and pathological conditions. They harbour substances that mirror the content of their cell of origin [21] and have the capability to exhibit different biological functions on recipient cells via trafficking of different factors (i.e. nucleic acids, proteins, lipids) [22]. hESCs-derived exosomes have been reported to reprogram hematopoietic stem/progenitor cells [23], however, the possibility that these hESCs-derived exosomes could be the main effectors of the hESCs-mediated anti-tumor effect and reprograming of malignant cancer has not been yet fully investigated. We hypothesized that hESCs-derived exosomes contain embryonic stem cell-specific reprogramming factors that can be delivered to target cancer cells, which subsequently revert to a benign phenotype. To address this hypothesis, we firstly examined the effect of the hESCs-CM on cancer cell growth in vitro and its effect on tumorigenicity in vivo. We then investigated the effects of hESCs-derived exosomes on tumor cells. We found that hESCs-CM contains factors that not only induce an anti-proliferative and pro-apoptotic effects on cancer cells in vitro but also inhibit the tumorigenicity of cancer cells both in vitro and in vivo. We also showed that hESCs-derived exosomes were efficiently internalized. Following internalization, exosomes were able to transfer their cargo to target cancer cells and induce a dose-dependent increase in SOX2, OCT4 and Nanog proteins, with a concomitant dose-dependent decrease in the proliferation and increase in the apoptosis of cancer cells. Moreover, exposure of cancer cells to hESCs-derived exosomes decreased tumor size when cancer cells were transplanted in a xenograft model. This study shows for the first time that hESCs-derived exosomes play an important role in the tumor suppressive activity exhibited by hESCs through the transfer of their cargo into target cells, which reprograms malignant cancer cells towards a benign phenotype with subsequent decrease in cancer cells tumorigenicity. The results of this study have implication in the understanding of cancer behavior, and may pave the way for the design of new anticancer therapies.

## Materials and Methods

Mice were used in compliance with McGill University Health Centre Animal Compliance Office (Protocol #: 2012–7280)

### Human embryonic stem cell cultures and preparation of conditioned medium (hESCs-CM)

The WA01 and WA09 cell lines (WiCell) were cultured on matrigel pre-coated plates (BD Biosciences) in mTeSR1 medium (Stemcell Technologies). For passage, undifferentiated hESCs colonies were incubated with dispase at 37°C for 10 min and were subsequently removed mechanically. After 3 washes, hESCs were replated on matrigel-coated plate. Cells were maintained at 37°C in a 5% CO_2_ humidified atmosphere. hESCs used in these experiments were at less than passage 35. For conditioned medium recovery, hESCs were plated on growth factor-reduced matrigel (BD Biosciences) in mTeSR1 media. Supernatant was collected when cells reached 60–90% confluence, centrifuged at 300 g for 10 min to pellet cells, then cleared of any remaining debris by centrifugation at 2,000 g for 20 min. Supernatant was filtered through a 0.2 μm filter to remove particles larger than 200 nm, aliquoted and stored at -80°C until use. To test for contamination or cell carry-over, aliquots of the hESCs-CM were put in a culture plate and incubated at 37°C, 5% CO2 for several weeks.

### Isolation and labeling of exosomes from hESCs-CM

Exosomes were prepared by differential centrifugation. Briefly, contaminating cells were removed by centrifugation at 300 g for 10 min, followed by serial centrifugation at 1,200 g for 20 min, and 10,000 g for 30 min to remove debris and large vesicles. After filtration (0.2 μm), exosomes were pelleted by ultracentrifugation (Beckman ultracentrifuge, Beckman Coulter) at 100,000 g for 70 min. The pelleted exosomes were washed twice in PBS, re-suspended in PBS, and stored at -80°C until used. The purity of the exosomes was verified, as stated below, by Western blot for selective exosome markers (i.e. SSEA4, CD63 and GM130). The supernatant of the pelleted exosomes was used as a control in functional tests.

For exosome uptake analysis, the purified exosomes were labeled with PKH26 red fluorescent probe according to manufacturer’s instructions (Sigma-Aldrich). Briefly, exosome pellets were suspended in Diluent C and mixed with equal volume of the stain solution (4 μl PKH26 in 1 ml Diluent C). After 4 min incubation, the reaction was stopped by adding an equal volume of 1% BSA. Labeled exosomes were washed twice with PBS and recovered by ultracentrifugation at 100,000 g for 70 min.

### Electron microscopy (EM) and size distribution analyses of exosomes

Exosomes were fixed in 2% paraformaldehyde (w/v) in 200 mM phosphate buffer (pH 7.4), overlaid on a Formvar carbon-coated grid (FCF400-NI-50; Electron Microscopy Sciences, Hatfield, PA, USA) and left to dry. After 3 washes in phosphate buffer, the exosomes were further fixed in 1% glutaraldehyde for 5 min, washed in distilled water, and stained with aqueous uranyl oxalate (pH 7) for 5 min. The exosomes were then stained with saturated aqueous uranyl acetate, and samples were embedded in 0.4% uranyl acetate and 1.8% methylcellulose on ice for 10 min. Excess liquid was then absorbed with a Whatman filter (Sigma-Aldrich). The grids were dried at room temperature for 5 min before analyses. Exosome samples were visualized with the CM100 electron microscope (Philips, Eindhoven, The Netherlands).

In parallel, an aliquot of exosome samples was run on a Nanosight NS500 system (Nanosight Ltd., Amesbury, UK), and size distribution was analyzed using the NTA 1.3 software.

### Cancer cell lines and culture conditions

Human mammary carcinoma cell lines MCF-7 and MDA-MB-231, and human colorectal adenocarcinoma Colo-320 and HT-29 (ATCC, VA, USA) were maintained in DMEM/F12 supplemented with 10% fetal bovine serum and penicillin/streptomycin (Wisent, Saint-Bruno, Canada), which had been filtered through 0.2 μm filters. Cells were treated with hESCs-CM or exosomes (hESCs-Exo), with medium change every day, in humidified atmosphere containing 95% air and 5% CO_2_ at 37°C.

### Exosomes internalization analyses

10 μg of PKH26-labeled exosomes was added to 5,000 cells cultured in 8-well chamber slides (VWR). After 12 h incubation, cells were washed, and fixed for 10 min with Paraformaldehyde 4%. The slides were mounted with coverslip in VECTASHIELD Mounting Medium with DAPI (Vector Laboratories). Stained cells were visualized using an LSM780 confocal microscope (Zeiss).

### Cell proliferation assessment

Cell proliferation was assessed using Alamar Blue (Thermo Scientific) and CFSE (Invitrogen) labeling following manufacturer’s instructions. Briefly, the assays were performed at the end of the culture experiments. Alamar Blue was added to culture medium (100 μl/ml of medium) and incubated for 6 h. Fluorescence was monitored at 530–560 nm excitation wavelength and 590 nm emission wavelength in a 96 well plate using a fluorescence multi-plate reader (FLUOstar OPTIMA, BMG LABTECH). For CFSE labeling, cultures were treated with 1 μM CFSE in pre-warmed PBS for 15 min at 37°C. Labeling solution was replaced by pre-warmed culture medium, and cells were cultured for 30 min at 37°C to allow acetate hydrolysis. Cells were washed, incubated for different time points and analyzed using a FACScan flow cytometer (Becton Dickinson).

### Cell cycle analysis

To analyze the percentage of cells in each cell cycle stage, treated and control cells were fixed in absolute ethanol for 2 h. and were permeablized with 0.1% Triton X-100. Cells were labeled with propidium iodide (PI), and were acquired with a FACScan flow cytometer. Analyses were performed using FlowJo software (Treestar).Cell viability assessment using Annexin V / Propidium iodide labelling

For the analysis of apoptosis, dissociated cells were resuspended in AnnexinV binding buffer, and stained with FITC-Annexin V (PharMingen). Just before cell acquisition, 5 μl of propidium iodide was added. Cells were acquired within 1 h. in a FACScan flow cytometer.

### Soft agar colony formation (anchorage independent cell growth) assay

Anchorage-independent cell growth was determined by analyzing the formation of colonies in soft agar. Soft agar assays were conducted in 12-well plates in semi-solid media. After trypsinization, 5,000 cells were suspended in 10% FBS-supplemented DMEM medium containing 0.3% noble agar. This suspension was layered on top of 0.8% agar-containing medium. Colonies (containing at least 50 cells) were scored and photographed after 3–4 weeks of culture under an inverted microscope Evos XL AMG (Fisher Scientific). The size of all colonies in a given culture condition was determined using ImageJ Software. The values obtained were then categorized to compare one culture condition to another.

### Quantitative real-time PCR

All primers were designed to flank individual exons and tested by PCR of RT^+^ and RT^-^ control extracts. Total RNA was isolated using TRIzol reagent (Invitrogen). Reverse transcription (RT) was done on 1 μg of cellular total RNA or 100 ng of exosomal RNA using the MML-V reverse transcriptase (Invitrogen). Quantitative real-time PCR (qPCR) was performed using the Platinum SYBR Green SuperMix (Invitrogen) and an ABI Prism 7500 Real-Time PCR apparatus (Applied Biosystems). Primer sets used were as follows: human GAPDH: forward primer 5’-TGACAACTTTGGTATCGTGGAAGG-3’; reverse primer 5’-AGGGATGATGTTCTGGAGAGCC-3’; human SOX2: forward primer 5’-CATGAAGGAGCACCC-GGATT-3’; reverse primer: 5’-TAACTGTCCATGCGCTGGTT-3’; Human OCT4 forward primer 5’-CTGGGGGTGATACTTGAGTGA-3; reverse primer 5’-TCCCAGGGTGATCCTCTTCT-3’, Human NANOG: forward primer 5’-AGCAGATGCAAGAACTCTCCA-3’; Reverse primer: 5’-TAAAGGCTGGG-GTAGGTAGG-3’. GAPDH was used as an internal standard for data calibration. The 2^-ΔΔCt^ formula was used for the calculation of differential gene expression.

### Immunofluorescence staining

For immunocytofluorometry, cells were fixed in 4% paraformaldehyde (w/v) for 15 min, and permeabilized in PBS/0.3% Triton X-100 for another 15 min. Cells were washed with PBS, and blocked in 2% BSA for 1 h. Primary antibodies against Nanog, Oct4 and Sox2 (all from Abcam) were added to cells at 1:250 dilution in 2% BSA and incubated overnight at 4°C. Cells were washed with PBS and labeled for 1 h with fluorophore-conjugated secondary antibodies (1:500 dilution; Alexa Fluor 594-donkey anti-rabbit, Alexa Fluor 594 donkey-anti-mouse, Oregon Green 488-goat anti-rabbit). Cells were washed with PBS and the slides were mounted on coverslips with DAPI-containing mounting medium (Vector Laboratories). Cells were visualized using an LSM780 confocal microscope.

### Western blot

Total protein extracts were prepared in the Complete Mini protease inhibitor cocktail solution (Roche Diagnostics) and sonicated. Proteins contents were quantified using the Bradford reagent, resolved in Laemmli buffer by SDS-PAGE and transferred to a Nitrocellulose Blotting Membrane (Pall). Membranes were blocked for 1 h in 5% non-fatty milk in TBS containing 0.05% of Tween-20 and incubated overnight with primary antibodies (mouse anti-OCT4, rabbit anti-Sox2, rabbit anti-Nanog (all from GenTex), mouse anti-GM130, anti-β-actin and anti-CD63 (all from Abcam), anti-SSEA4 (BD Biosciences), and anti-α-tubulin (Sigma)). Membranes were treated with corresponding horseradish peroxidase-conjugated secondary antibodies (Sigma) and developed using the Immobilon Western (Millipore).

### In vivo tumor formation

Five-week-old female NOD-SCID mice (Jackson Laboratory) were used in compliance with McGill University Health Centre Animal Compliance Office (Protocol 2012–7280). Control and treated cells were harvested by trypsinization and washed twice with HBSS. Mice were injected subcutaneously with 2 million cells in 200 μl HBSS/Matrigel mixture (VWR). Mice were injected in both flanks to reduce the number of animals used in compliance with the “Three Rs” principles of the Animal Care Committee of our institution. By using this strategy, every treatment group was analyzed from 4 to 6 times. The animals were monitored for activity and physical conditions every day. Mice were euthanized one month post-injection. The resulting xenotransplants were photographed, their diameters were recorded with a caliper and their volumes were estimated using the following formula V = a × b^2^× (π/6) (where a = major diameter; b = minor diameter and V = volume). Animals were euthanized by cervical dislocation when the tumor was ≥ 1 cm diameter. Images of the resulting xenotransplants were acquired and processed as indicated below.

### Immunohistochemistry labelling procedures

Mice xenotumors were collected, fixed in 10% buffered formalin, embedded in paraffin, and stained with hematoxylin and eosin (H&E) according to standard protocols or processed for immunohistochemistry. Briefly, 5 μm tissue sections were dewaxed in xylene and rehydrated with distilled water. After antigen unmasking, and blocking of endogenous peroxidase (3% hydrogen peroxide), the slides were incubated with mouse anti-cytokeratin 7 (CK7, DAKO) and rabbit anti-Ki67 (Ventana) antibodies. Labeling was performed using iView DAB Detection Kit (Ventana) on the Ventana automated immunostainer. Sections were counterstained lightly with Hematoxylin before mounting.

### Statistical analysis

Statistical differences were analyzed using Student’s *t*-test for unpaired samples. An analysis of variance (ANOVA) followed by the Dunnett test was used for multiple comparisons with one control group. The criterion for significance was set at P value < 0.05.

## Results

### hESCs-CM induces an anti-proliferative and pro-apoptotic effects on cancer cells in vitro

**Fig 1:**
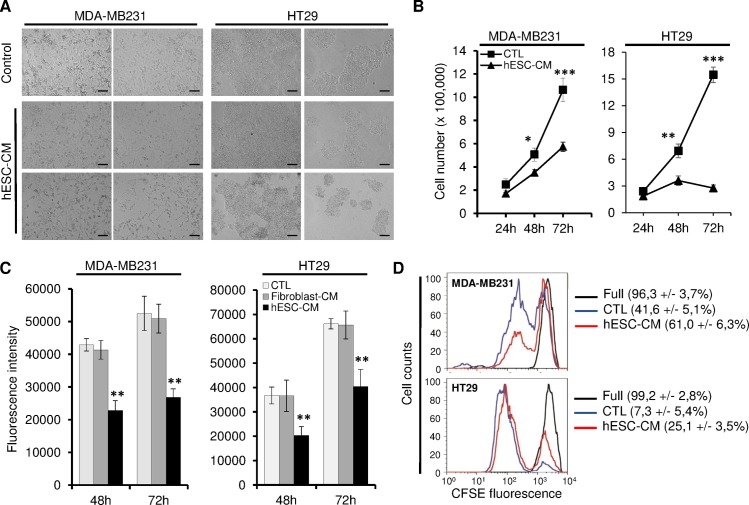
hESCs-CM decreased cancer cells growth. MDA-MB231 and HT29 cells were cultured for 3 days in control medium or hESCs-CM, and cells were analyzed for their growth potential. (A) Bright field pictures of cell cultures at 3 days post-treatments. Note the significant reduction in cell density in cultures maintained in hESCs-CM. Scale bar: 50 μm. (B) 100,000 cells were plated and their number was followed for the 3 days of culture period. Values are cells counts presented as mean ± SD (n = 3 independent cultures, *P < 0.05, **P < 0.01, ***P < 0.001). (C) The metabolic activity following 2 and 3 days treatment duration. Cultures were incubated for 5 h with Alamar Blue and data acquired by spectrofluorometry. Data are presented as mean ± SD and are representative of 3 independent experiments (**P < 0.01). (D) CFSE load dilution in cultures at 3 days. Full refer to CFSE loading at the beginning of the culture period. Numbers in brackets are the percentages of fully CFSE-loaded cells (cells that did not divide yet). Data are mean ± SD (n = 3 independent experiments, P < 0.05 in MDA-MB231 cultures and P < 0.01 in HT29 cultures).

To analyze the effect of hESCs-CM on cancer cell growth, we used four cancer cell lines (Colo-320, MCF-7, MDA-MB-231 and HT29). Cells were cultured in either hESCs-CM (mTeSR1 medium collected from hESCs cultures), or control medium (mTeSR1 medium not exposed to hESCs) for 3 days with daily medium change. In control medium, all tested cell lines grew rapidly and reach almost 90% confluence by the third day of culture. In contrast, cells cultured in hESCs-CM exhibited a slower growth and failed to reach full confluence (Fig 1A and S1 Fig). This observation was confirmed when we compared cell counts following treatments. Cell growth kinetic curve showed that, as early as 48 h after the beginning of the exposure to hESCs-CM, cells grew almost 30 to 50% less when compared to those maintained in control medium. This decreased growth pattern showed an even further decrease at 72 h post-treatment (29 to 80% cell growth reduction, Fig 1A and 1B and S1 Fig). Interestingly, in the case of MDA-MB-231 cells, hESCs-CM not only dramatically inhibited their growth but also altered their morphology (Fig 1A). Immunofluorescence staining showed that hESCs-CM-treated cells down-expressed vimentin, suggesting a loss of their mesenchymal phenotype (S2 Fig). The effects of hESCs-CM on the growth of cancer cells was further analyzed by assessing cells metabolic activity (i.e Alamar blue labeling) and cell division (i.e. CFSE staining). Notably, hESCs-CM treatments decreased the metabolic activity of treated cancer cells, and their division rhythm as shown by the delay in diluting their CFSE probe load (Fig 1C and 1D). To rule-out the possibility that the effects on cell growth observed with hESCs-CM was a side effect due to either growth factors and nutrients depletion of the culture medium, or to the cytostatic effect of putative cell metabolic by-products, we cultured MDA-MB-231 and HT29 cells in human fibroblasts-derived conditioned medium (Fibro-CM). As opposed to hESCs-CM, Fibro-CM did not affect cell growth even at 3 days post-treatment, suggesting that the observed effects on cell growth were specific to hESCs-CM (Fig 1C).

**Fig 2:**
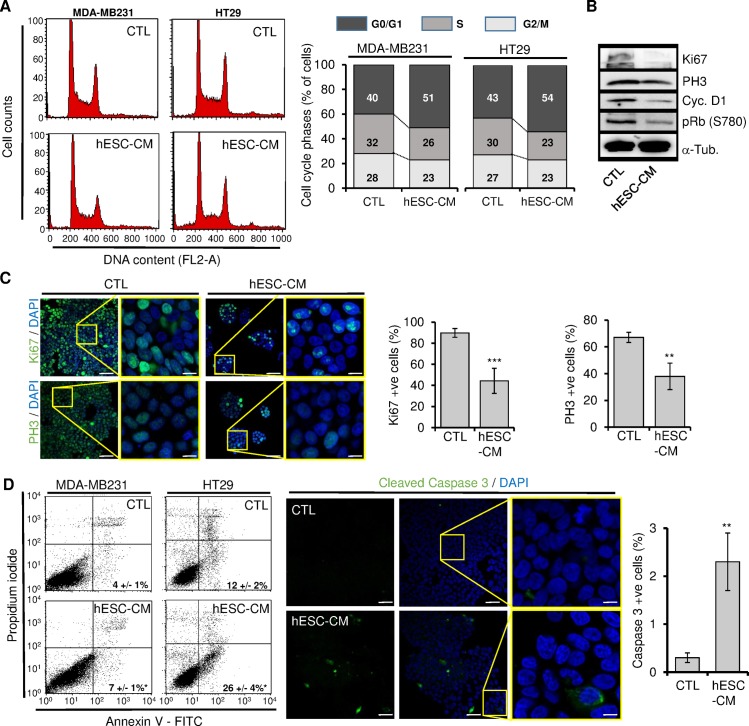
hESCs-CM inhibited cell cycle progression of cancer cells and initiated a pro-apoptotic program. MDA-MB231 and HT29 cells were cultured for 3 days in control medium or hESCs-CM, and cells were analyzed for their proliferation (A-C) and cell viability (D). (A) Control and hESCs-CM-treated cells were loaded with propidium iodide and analyzed for their progression in the cell cycle. Barre plots display the data of 3 independent experiments. Note that hESCs-CM-treated cells progress slowly through the G1 phase. (B) Cells were analyzed by Western blot for the expression of cell cycle regulatory proteins. alpha-Tubulin (α-Tub.) was used as a proteins loading control. (C) Cells were analyzed by immunocytofluorometry for the expression of Ki67 and phosphor-histone 3 (PH3). The graphs display the raw data. Values are mean ± SD of positive cells (n = 3 independent cultures, **P < 0.01, ***P < 0.001). Scale bars: 50 and 15 μm in low and high magnification, respectively. (D) (Left) Cells were analyzed for apoptosis following labeling with Annexin V and loading of propidium iodide (PI). Apoptotic cells (Annexin V positive and PI negative) were scored and their percentages were shown (bottom right corner, n = 3 independent cultures, *P < 0.05). (Right) Parallel cultures were plated on chamber slides and analyzed for the cleavage of caspase 3. Values are mean ± SD of positive cells (n = 3 independent cultures, **P < 0.01). Scale bars: 50 and 10 μm in low and high magnification, respectively.

We then wanted to verify if the above-observed effects might be due to reduced proliferation and/or increased cell death. We analyse cell cycle progression of cells cultured in both control medium and hESCs-CM. As compared to cells maintained in control medium, those grown in hESCs-CM accumulated more in the G0/G1 phase, and less in S and G2/M phases (Fig 2A). These observations were consistent with down-expression of the proliferation markers (Ki67, phosphor-histone 3 (PH3), cyclin D1, and phosphorylated retinoblastoma protein (pRb) in hESCs-CM-treated cells (Fig 2B and 2C). In parallel, we assessed cell viability in these cultures by using AnnexinV/PI staining coupled with flow cytometry analyses. When compared to control medium-treated cells (4 ± 1% and 12 ± 2% apoptotic cells in MDA-MB-231 and HT29, respectively), those treated with hESCs-CM displayed increased apoptosis (7 ± 1% and 26 ± 4% in MDA-MB-231 and HT29 cells, respectively) (Fig 2D). In parallel, hESCs-CM-treated cells expressed more activated caspase 3 than those exposed to control medium (Fig 2D). Taken together, these results indicate that hESCs-CM induce growth arrest of cancer cells by reducing their proliferation and promoting apoptosis.

### hESCs-CM inhibits the tumorigenic potential of cancer cells

**Fig 3:**
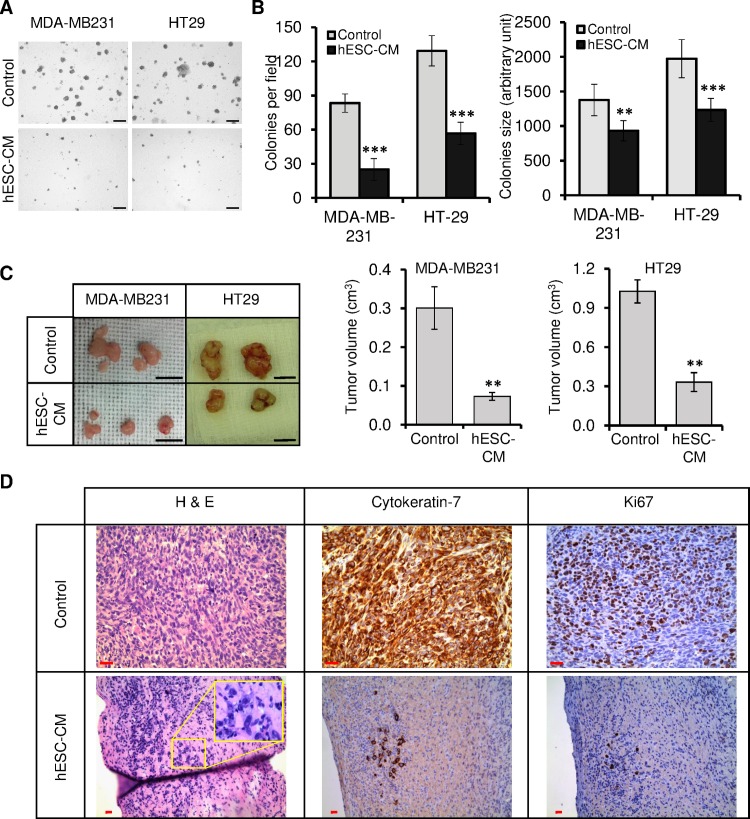
hESCs-CM inhibited the oncogenic potential of cancer cells in vitro and in vivo. MDA-MB231 and HT29 cells were cultured for 2 weeks in control medium or hESCs-CM. (A and B) Cells were grown in soft agar for another 2 weeks to analyze their anchorage-independent growth. (A) Bright field pictures. Note the decrease of colony sizes and numbers when cells were exposed to hESCs-CM. Scale bar: 200 μm. (B) (Left) The graph represents the number of colonies counted per field. (Right) The graph represents the size of the colonies obtained. Colonies were measured using ImageJ software. Data are presented as mean ± SD (n = 3 independent experiments, **P < 0.01, ***P < 0.001). (C and D) Cells treated as in (A) were injected subcutaneously in NOD/SCID mice. (C) 4 weeks after injection, xenograft were photographed and their volumes were calculated. Values are mean ± SD, (n = 4–6 xenotransplants, **P < 0.01). Scale bar: 1 cm. (D) Formalin-fixed paraffin-embedded xenotransplant samples were processed for H&E staining, or immunolabeled with antibodies against cytokeratin-7 and Ki67 (tumors obtained with MDA-MB231 cells are shown). Scale bar: 40 μm.

The potential to perform anchorage-independent growth is a hallmark of transformed cells. To test whether HT29 and MDA-MB-231 cells lost their characteristics of transformed cells in vitro upon treatment with hESCs-CM, we treated them with control medium and hESCs-CM for 2 weeks with continuous medium replacement, and at the end of the second week, we analyzed their ability to form colonies in soft agar substrate (Fig 3A and 3B). We observed that the incidence of colony formation was reduced in hESCs-CM-treated cultures when compared to control medium-treated cultures (Fig 3A and 3B). Also, colony size analyses showed that cells cultured in hESCs-CM gave rise to smaller colonies when compared to those generated by cells grown in control medium (Fig 3A and 3B).

To determine whether hESCs-CM-treated cancer cells loss their ability to form tumors in vivo, NOD/SCID mice were injected subcutaneously with cancer cells treated as in Fig 3A. Mice were followed-up for tumor growth until the size of the mass reached 1 cm in diameter. Mice injected with control medium-treated cancer cells developed bigger tumors that those generated with cancer cells treated with hESCs-CM (Fig 3C).

Histopathological analyses of all excised tumors confirmed they were adenocarcinomas. However, while tumors obtained with cells treated with control medium displayed high mitotic index and CK-7 staining (77–90%), those tumors obtained with cells exposed to hESCs-CM showed only focal positive clones (Fig 3D). All together, these results suggest that hESCs-CM contains factors that inhibit the tumorigenic potential of cancer cells both in vitro and in vivo.

### hESCs-derived exosomes (hESCs-Exo) are efficiently internalized by target cancer cells

**Fig 4:**
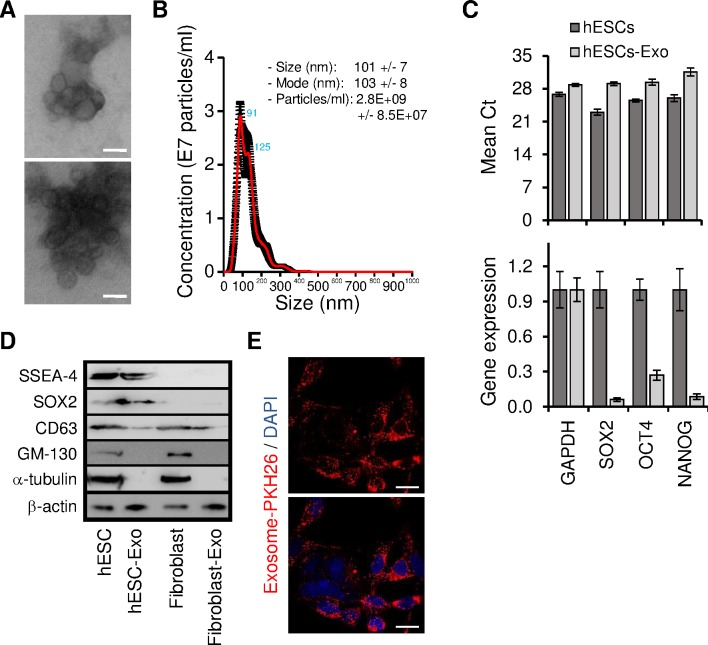
Cancer cells internalized efficiently hESCs-Exo. (A) Exosomes were isolated as described under Material and Methods. Representative micrographs of TEM show small vesicles of approximately 50–120 nm in diameter. Scale bar: 100 nm. (B) NanoSight analyses of samples prepared as in (A). The size was centered around 101 nm in diameter. Data are expressed as concentration average (red line) of 3 exosome preparations. (C) qPCR analyses for the expression of pluripotency transcription factors SOX2, OCT4 and NANOG transcripts in hESCs and hESCs-Exo. Upper panel; data are expressed as threshold cycle (Mean Ct). Lower panel; data were normalized to the level of GAPDH, and the levels of SOX2, OCT4 and NANOG transcripts expression in hESCs were set at 1. Results are presented as mean ± SD (n = 2 independent experiments repeated in triplicates). (D) Proteins isolated from cells (hESCs and fibroblasts) or exosomes (hESCs-Exo and fibro-Exo) were analyzed by Western blot for the expression of specific hESCs and exosomes markers. (E) Confocal microscopy monitoring of PKH-26-labeled (red dots) exosome uptake *in vitro* into MDA-MB231 cells (12 h incubation). Note that exosomes are uniformly dispersed in the cytoplasm and tended to form aggregates in the perinuclear regions. Similar results were obtained with HT29 cells. Scale bar: 10 μm.

Exosomes have the capability to exert different biological effects on target cells, by transferring their contents into their target cells [21, 22]. Undifferentiated hESCs produce a significant amount of exosomes [24–26] and these hESCs-derived exosomes (hESCs-Exo) have also been reported to reprogram hematopoietic stem/progenitor cells via the horizontal transfer of mRNA and proteins [23]. We hypothesized that hESCs-Exo might be also involved in the reprogramming of malignant cancer cells to a more benign phenotype and thus be the effectors of the observed anti-tumor action of the hESCs-CM. For this purpose, we isolated exosomes from hESCs and confirmed their identity physically and phenotypically (Fig 4). Electron microscopy analyses showed that isolated hESCs-Exo were rounded structures with a size of approximately 30–100 nm (Fig 4A). In parallel, exosomes tracking analyses using a Nanosight system showed an average size of 101 +/- 7 nm (Fig 4B). This is in the range of the known exosome size [27]. Exosomes displayed specific markers that distinguish them from other cellular microvesicles [27–29] as shown by Western blot analyses (Fig 4D). Isolated exosomes expressed CD63 (a commonly used marker of exosomes) and the plasma membrane associated protein, β-actin but did not express the Golgi membrane bound protein GM130, suggesting that hESCs-Exo preparations were not contaminated with other vesicles or cellular components (Fig 4D). Notably, hESCs-Exo expressed pluripotency transcription factors transcripts and proteins (Fig 4C and 4D and S3 Fig). All transcripts analyzed (i.e. SOX2, OCT4 and NANOG) were expressed at lower levels in hESCs-Exo when compared to their expression levels in hESCs (Fig 4C).

In order to deliver their cargo and to exert their effects on target recipient cells, exosomes need to be uptaken by these cells. To study the internalization of hESCs-Exo, exosomes were labeled with the fluorescent probe (PKH-26) and added to cancer cells cultures. We found that after 12 h of incubation, cancer cells efficiently internalized the hESCs-derived exosomes (Fig 4E). Internalized exosomes were uniformly dispersed in the cytoplasm and tended to form aggregates in the perinuclear regions.

### hESCs-Exo dose-dependently decrease the proliferation and increase the apoptosis of cancer cells

**Fig 5:**
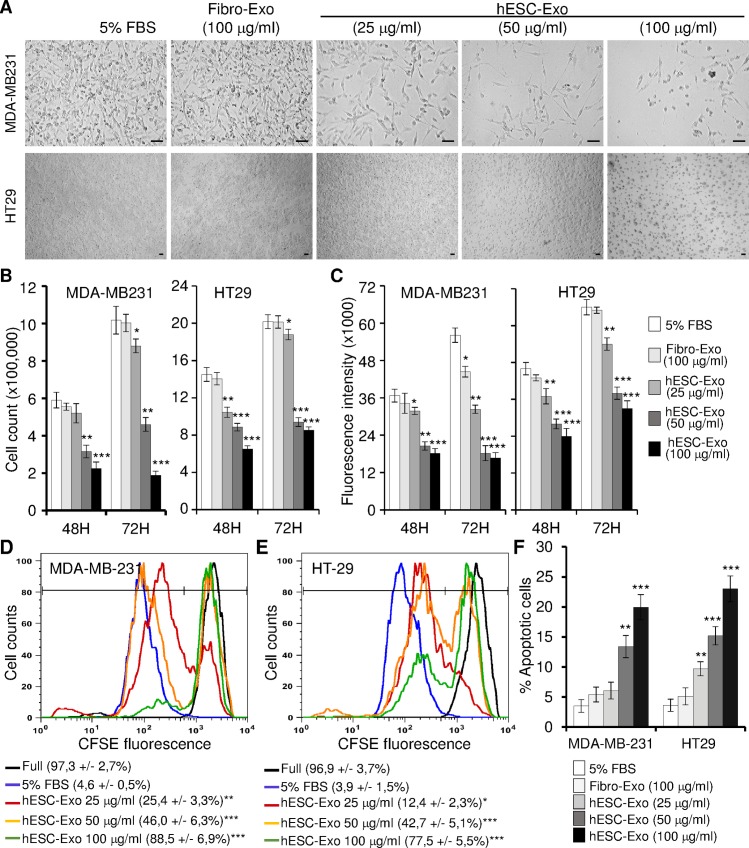
hESCs-Exo decreased cancer cell proliferation and increased cancer cell death. MDA-MB231 and HT29 cells were cultured for 3 days in control medium (5%FBS), or with exosomes derived from fibroblast-CM (Fibro-Exo) or hESCs-CM (hESCs-Exo), and cells were analyzed for their growth potential (A-E), and apoptosis (F). (A) Bright field pictures of cell cultures at 3 days post-treatments. Note the significant dose-dependent reduction in cell density in cultures maintained in hESCs-Exo. Scale bar: 50 μm. (B and C) note that the legend is the same for all graphs: (B) 100,000 cells were plated and their number was counted after 2 and 3 days of culture. Values are presented as mean ± SD (n = 3 independent cultures, *P < 0.05, **P < 0.01, ***P < 0.001). (C) The metabolic activity following treatment for 2 and 3 days. Cultures were incubated for 5 h with Alamar Blue and data acquired by spectrofluorometry. Data are presented as mean ± SD and are representative of 3 independent experiments (*P < 0.05, **P < 0.01, ***P < 0.001). (D and E) CFSE load dilution in cultures at 3 days post-treatments. Full refer to CFSE loading at the beginning of the culture period. Numbers in brackets are the percentages of fully CFSE-loaded cells (cells that did not divide yet). Data are mean ± SD (n = 3 independent experiments, *P < 0.05, **P < 0.01, ***P < 0.001). (F) Cell apoptosis analyses following labeling with Annexin V and loading of propidium iodide (PI). Apoptotic cells (Annexin V positive and PI negative) were scored and their percentages were shown (n = 3 independent cultures, **P < 0.01, ***P < 0.001).

To investigate the effects of hESCs-Exo on cancer cells, MDA-MB-231 and HT29 cells were cultured in mTeSR1 medium supplemented or not with increasing amounts of hESCs-Exo. Cells were analyzed at two time-points (i.e. 48 h and 72 h) after the beginning of the treatments. When cells were treated with hESCs-CM without exosomes, they grew rapidly. In contrast, cells maintained in hESCs-Exo-containing medium displayed slower growth and failed to reach full confluence (Fig 5A). hESCs-Exo effects were dose-dependent reaching a maximum at an exosome load of 50–100 μg/ml (which correspond to 4.8–9.6e+07 particles/ml). The observed effects on cell growth were confirmed when we compared cell number counts (Fig 5B), cell metabolic activity (Alamar blue labeling) (Fig 5C) and cell division (CFSE load dilution) (Fig 5D and 5E). Indeed, hESCs-Exo treatments dose-dependently decreased cancer cell number and metabolic activity, and slowed their cell division potential (Fig 5B–5E). To rule-out the possibility that the observed effects on cell growth were due to an artefactual bias of the exosomes particles, the same analyses were performed by using exosomes collected from human fibroblasts (Fibro-Exo). As opposed to hESCs-Exo, Fibro-Exo did not show any effect on cancer cell growth even at the highest exosome load tested (i.e. 100 μg/ml) and the longest treatment period (i.e. 3 days) (Fig 5A–5C), suggesting that the observed effects on cell growth were specific to hESCs-Exo.

In parallel, we assessed cell viability in these cultures by flow cytometry analyses using AnnexinV/PI labeling. When compared to the control hESCs-CM without exosomes, cells treated with hESCs-Exo displayed a dose-dependent and significant increase of apoptotic annexin V-positive cells. Also in this case, Fibro-Exo did not affect cancer cells behavior (Fig 5F). Altogether, these data indicate that hESCs affect cancer cell growth by inhibiting cell proliferation and promoting cell death, mainly via factors carried as cargo in the exosomes.

### hESCs-Exo treatments reduce the tumorigenic potential of cancer cells

**Fig 6:**
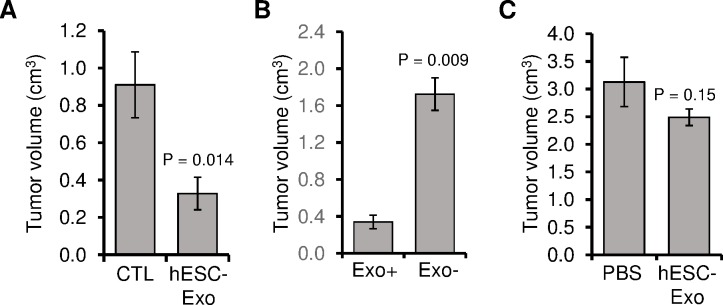
hESCs-Exo inhibited the oncogenic potential of cancer cells. (A) HT29 cells were cultured for 2 weeks in exosome free medium (CTL) or hESCs-Exo-containing medium (hESCs-Exo), and were injected subcutaneously in NOD/SCID mice. Three weeks after injection, xenograft volumes were calculated. (B) HT29 cells were exposed to PKH26-labeled hESCs-Exo. After 2 h incubation, cells were sorted based on hESCs-Exo internalized (PKH26 +ve vs. PKH26 –ve). Both pools of sorted cells were injected subcutaneously in NOD/SCID mice. Three weeks after injection, xenograft volumes were calculated. (C) HT29 cells were injected subcutaneously. When tumors appeared (~3 weeks post injection), they were injected in the tumor masses with either HBSS or hESCs-Exo (~24 μg; 10 μl) every second day for 3 weeks, and the xenograft volumes were calculated. Values are mean ± SD, (n = 4–6 xenotransplants, P values are shown).

To evaluate whether hESCs-Exo are able to affect the tumorigenic behavior of cancer cells, HT29 cells were cultured for 2 weeks in control media or hESCs-Exo-containing media, then were injected subcutaneously in NOD/SCID mice, which were followed for tumor growth (Fig 6). Mice injected with control medium-treated cancer cells developed bigger tumors than those generated with cancer cells treated with hESCs-Exo-containing medium (64% tumor size reduction with cells treated with hESCs-Exo, P = 0.014) (Fig 6A).

As we observed that cancer cells did not uniformly internalize hESCs-Exo (Fig 4C), we hypothesized that only cancer cells that uptake efficiently hESCs-Exo would display reduced tumorigenic behavior. To test this assumption, HT29 cells were exposed to PKH26-labeled hESCs-Exo. After 2 h of incubation, cells were FACS-sorted based on their hESCs-Exo internalization (i.e. PKH26 positive vs. PKH26 negative cells). Both pools of sorted cells were injected subcutaneously in NOD/SCID mice, which were followed for tumor growth. Mice injected with PKH26 positive cancer cells (i.e. hESCs-Exo positive) developed minimal tumor masses when compared to those generated with PKH26 negative cancer cells (i.e. hESCs-Exo negative) (81% tumor size reduction, P = 0.009) (Fig 6B).

In another set of experiments, HT29 cells were injected subcutaneously in NOD/SCID mice. Once tumor masses appeared (~2 weeks), mice were injected in the mass with either HBSS or hESCs-Exo (~24 μg per site) every second day for 2 weeks to test the effect of the hESCs-Exo in vivo. Although not statistically significant, tumors that were treated *in situ* with hESCs-Exo were smaller than those treated with the vehicle (HBSS) (20% tumor size reduction, P = 0.15) (Fig 6C). Taken together, these data showed that hESCs-Exo has the potential to reduce cancer cells tumorigenicity.

### hESCs-Exo transfer their cargo to target cancer cells

**Fig 7:**
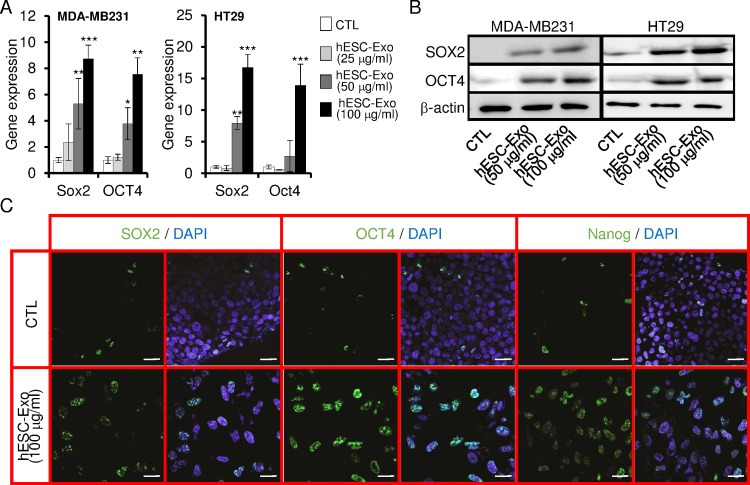
hESCs-Exo induced the expression of hESCs markers in cancer cells. Cells were treated with exosome-free medium (CTL) or hESCs-Exo medium, and analyzed for the expression of hESCs markers. (A) qPCR analyses for the expression of SOX2 and OCT4 transcripts. Data were normalized to GAPDH, and the level of transcripts expression in control medium was set at 1. Results are presented as mean ± SD (n = 3 independent experiments, *P < 0.05, **P < 0.01, ***P < 0.001). (B and C) Parallel cultures were analyzed for the expression of hESCs protein markers by Western blot (B) and cytofluorometry (C). In (B), β-actin is used as calibrator for proteins loading. Scale bar: 20 μm.

We observed that cancer cells efficiently internalized hESCs-Exo (Fig 4E), and that these exosomes contained both mRNA and proteins of specific hESCs pluripotency markers (i.e. SOX2, OCT4, NANOG, SSEA4) (Fig 4C and 4D). Therefore, we verified if these pluripotency markers would be transfered to target cancer cells via hESCs-Exo. The pluripotency transcription factors are normally present in minute amount in cancer cells (Fig 7). We treated both HT29 and MDA-MB231 cancer cells with increasing amounts of hESCs-Exo for 3 days. The exposure to hESCs-Exo induced a dose-dependent increase in SOX2, OCT4 and Nanog proteins (Fig 7B and 7C). Increased expression may be due to direct transfer of hESCs-Exo cargo and/or de novo translation of mRNA. Using RT-qPCR analyses, we observed that SOX2 and OCT4 transcripts were dose-dependently and significantly increased in both cancer cell lines following treatments with hESCs-Exo (Fig 7A). This finding suggests that the observed re-expression of hESCs pluripotency factors in cancer cells might perhaps be due to both direct transfer of hESCs-Exo cargo to cancer cells and to de novo translation of mRNA.

## Discussion

In the present study, we reported that conditioned medium and exosomes derived from hESCs inhibited the growth of cancer cells and reduced their tumorigenic potential both in vitro and in vivo. Also, we observed that hESCs-Exo induced re-expression of pluripotent stem cell markers at both mRNA and protein levels in target cancer cells, pointing to their possible role in the reprograming of malignant cancer cells toward a benign phenotype.

We showed that factors contained in hESCs-CM inhibited the growth of cancer cells by increasing apoptotic cell death, and by arresting cancer cells at the G1 phase of the cell cycle, with concomitant decrease of cells at the S and G2/M phases. These effects were not due to a side effect consequential to a depletion of of crucial growth factors and nutrients in the condition medium, or to the presence of cytostatic cell metabolic by-products, because these effects, were not observed when cancer cells were maintained in fibroblasts-derived conditioned medium or hESCs-CM depleted of exosomes.

The control of the cell cycle progression is essential for the maintenance of a proper proliferation balance. To prevent inappropriate cell proliferation, several cell cycle regulatory proteins play a role as gatekeeper [30] and control checkpoints that regulate cell cycle progression or cell cycle arrest. For instance, blocking cancer cells at the G1 phase will prevent the growth of the cancer. In this context, cyclin D1 activates a cascade that leads through the phosphorylation of the RB protein to the transcription of E2F-specific target genes responsible for the progression through the G1 phase [31]. We reported that the levels of cyclin D1 were decreased following cancer cells treatment with hESCs-CM, and this could have helped keeping RB hypophosphorylated thus preventing G1/S phase transition. In addition, we observed that phosphorylation at serine residue 10 in the histone H3 was drastically reduced. This histone modification is a crucial event for the onset of mitosis, and appears early in the G2 phase [32, 33].

Interestingly, hESCs-CM not only inhibited breast cancer cells MDA-MB-231 growth but also altered their morphology. Cell fate transition takes place during physiological and pathological processes, as well as during experimental manipulations (i.e. embryonic development, tumor progression and somatic cell reprogramming) [34]. This transition is characterized by the loss of certain phenotypical traits and the acquisition of others. Immunofluorescence staining performed in our experiments showed that about 90% of breast cancer cells had lost the expression of Vimentin concomitantly with a loss of their mesenchymal phenotype after exposure to hESCs-CM.

In the present study, treatment with hESCs-derived exosomes reproduced all the effects obtained with hESCs-CM. Exosomes are mediators of cell-to-cell communication that carry unique molecular signatures [35–37]. hESCs can produce a significant amount of exosomes that can exert different physiological effects both in vitro and in vivo, and could therefore be used as a new tool to reprogram malignant cancer cells via transfer of bioactive proteins, and nucleic acids into target cells [23, 38]. Herein, we report that hESCs-Exo transfer a full network of embryonic pluripotency transcription factors, not just a single pluripotency factor, into cancer cells thus leading to the reprograming of cancer cells to a more benign state by reducing cancer cells tumorigenesis. Our results strengthen the recent reported evidence that hESCs-Exo loaded with Lefty proteins have an inhibitory effect on Nodal signaling in aggressive melanoma cells [17].

Tumor growth and progression is driven by cancer stem cells that possess self-renewal ability [39], and the maintenance of CSCs is regulated by key embryonic stem cell transcription factors (i.e. SOX2, OCT4 and NANOG) [40–42]. These factors seem essential not only to maintain the stemness of hESCs (thus allowing embryonic development) but also they seem to have a role in the regulation of cancer features and tumorigenicity in the absence of major regulatory checkpoints [43]. To maintain their stemness, embryonic stem cells must keep a balanced network of core embryonic stem markers to preserve the equilibrium between cell proliferation, differentiation and apoptosis, as opposed to the over expression of a single factor that leads to loss of this equilibrium. It has been demonstrated that coordinated ectopic expression of OCT4, SOX2, KLF and c-MYC (OSKM) induces reprogramming of somatic cells pluripotency [44] while activation of individual core stem factors can contribute to tumorigenesis because the same pluripotency transcription factors are also integrated into different and separate networks that are associated with the formation of different cancer phenotypes [45–47]. These results confirm that the core stem transcription factors are integrated into a balanced network that control cell phenotypes and whose disruption might lead to the malignancy of normal cells.

CSCs aberrantly hijack some of these core embryonic stem cell markers and the imbalanced core stem signaling pathways drives CSCs population growth, which is eventually responsible for cancer progression [1, 42]. In this study, we showed that cancer cells treated with hESCs-derived exosomes re-express a network of core stem cell markers. Whether this expression is secondary to a transfer of their embryonic stem cell-specific cargo, or to a de novo stem marker gene expression induced in the target cells still has to be determined. The critical question that arises is how these core embryonic stem cell factors can reprogram cancer cells toward a benign phenotype with concomitant abrogation of their tumorigenic behavior. Our data suggest that hESCs-derived exosomes might exert their anti-oncogenic effects by enhancing the core stem cell markers expression to levels close to those found in ESCs, and possibly by reprograming target cancer cells to a pluripotent stage, restoring the normal development pathways. Early developmental signals naturally regulate proto-oncogenes so that their expression can be repressed [47] and therefore mimicking an early embryonic environment, with hESCs exosomes, might suppress some oncogenic phenotypes of cancer cells.

A differentiation hierarchy exists in both normal and cancer stem cell population. Stem cells show different degrees of differentiation potential and can produce a defined set of differentiated and specialized progeny [48]. Normal differentiation goes from a totipotent state to a pluripotent state, multipotent, unipotent, finally nullipotent, which results in cellular intermediates that are increasingly limited in terms of differentiation potential until the terminal differentiation. CSCs are ranked between multipotent and unipotent stage. However, during tumorigenesis, there is a loss of terminal differentiation with gain of uncontrolled proliferation and a recovery of differentiation ability ultimately accounting for tumor cellular growth, differentiation and heterogeneity. Here, we observed that hESCs-derived exosomes can reprogram cancer cells and partially suppress the malignant cancer phenotype. We hypothesize that hESCs-derived exosomes are able to reprogram a subset of cancer cells to the pluripotent or near-pluripotent state and to restore the balance of the core embryonic stem factors network in the target cancer cells. The pluripotency state imposed by the hESCs-derived exosomes could partially suppress the malignant cancer phenotype and allow reactivation of the blocked differentiation pathways, leading to differentiation into a benign phenotype with concurrent suppression of tumorigenicity.

The most striking finding of our research is that hESCs-derived exosomes can transfer their cargo to target cancer cells and subsequently reprogram these cancer cells toward a benign state. To our knowledge, this is the first demonstration that bestowing aspects of pluripotentiality to cancer cells through hESCs-derived exosomes results in the reprograming of cancer cells toward a benign phenotype with a simultaneous suppression of tumorigenicity. Our results mirror the finding from other studies, which proved that different types of human embryonic stem cells have the ability to inhibit cancer cell growth and tumorigenicity [9, 10, 49–51]. The identification of anti-tumorigenic factors involved in the effects described by us and by others may pave the way to their use as a stem cells cancer therapy. The first challenge will be to assess the oncogenic potential of cancer cells after the uptake of different amounts of these factors and define the specific factors involved, the pathways that are followed and the mechanisms that are implemented. When the specific tumour-suppressing factors secreted by the different types of stem cells are identified, the next challenge will be to determine the therapeutic dosage to which these factors can exert their pharmacological role as “reprogrammer” of cancer cells and their possible side effects on normal cells.

## Supporting Information

S1 Fig hESCs-CM decreased cancer cells growth. 100,000 Colo-320 and MCF-7 cancer cells were plated in control medium or hESC-CM for 3 days, and cells were analyzed for their growth potential. Bright field pictures of cell cultures at 3 days post-treatments. Note the significant reduction in cell density in cultures maintained in hESC-CM. Values are cells counts presented as mean +/- SD (n = 3 independent cultures, P < 0.05 when comparing control medium-treated cells to those treated with hESC-CM. Scale bar: 100 μm.
(TIF)

S2 Fig hESCs-CM downregulated the expression of vimentin in MDA-MB231 cells. Cells were plated in control medium or hESC-CM for 3 days, and were analyzed by immunocytofluorometry for the expression of vimentin. Scale bar; 25 μm.
(TIF)

S3 Fig hESCs-Exo carry pluripotency transcription factors. hESCs and hESCs-Exo isolated RNA were analyzed for the expression of pluripotency transcription factors SOX2, OCT4 and NANOG transcripts. Graphs display melt curves for the genes analyzed (n = 2 independent experiments repeated in triplicates).
(TIF)

## References

[1] Hendrix MJ, Seftor EA, Seftor RE, Kasemeier-Kulesa J, Kulesa PM, Postovit LM. Reprogramming metastatic tumour cells with embryonic microenvironments. Nat Rev Cancer. 2007;7(4):246–55. 10.1038/nrc2108 17384580

[2] Bailey CM, Kulesa PM. Dynamic interactions between cancer cells and the embryonic microenvironment regulate cell invasion and reveal EphB6 as a metastasis suppressor. Mol Cancer Res. 2014;12(9):1303–13. PubMed Central PMCID: PMCPMC4498260. 10.1158/1541-7786.MCR-13-0673 24836890 PMC4498260

[3] Joel M, Sandberg CJ, Boulland JL, Vik-Mo EO, Langmoen IA, Glover JC. Inhibition of tumor formation and redirected differentiation of glioblastoma cells in a xenotypic embryonic environment. Dev Dyn. 2013;242(9):1078–93. 10.1002/dvdy.24001 23780720

[4] Mintz B, Illmensee K. Normal genetically mosaic mice produced from malignant teratocarcinoma cells. Proc Natl Acad Sci U S A. 1975;72(9):3585–9.PubMed Central PMCID: PMCPMC433040. 1059147 10.1073/pnas.72.9.3585 PMC433040

[5] Illmensee K, Mintz B. Totipotency and normal differentiation of single teratocarcinoma cells cloned by injection into blastocysts. Proc Natl Acad Sci U S A. 1976;73(2):549–53.PubMed Central PMCID: PMCPMC335947. 1061157 10.1073/pnas.73.2.549 PMC335947

[6] Diez-Torre A, Andrade R, Eguizabal C, Lopez E, Arluzea J, Silio M, et al Reprogramming of melanoma cells by embryonic microenvironments. Int J Dev Biol. 2009;53(8–10):1563–8. 10.1387/ijdb.093021ad 19924629

[7] Lee LM, Seftor EA, Bonde G, Cornell RA, Hendrix MJ. The fate of human malignant melanoma cells transplanted into zebrafish embryos: assessment of migration and cell division in the absence of tumor formation. Dev Dyn. 2005;233(4):1560–70. 10.1002/dvdy.20471 15968639

[8] Kulesa PM, Kasemeier-Kulesa JC, Teddy JM, Margaryan NV, Seftor EA, Seftor RE, et al Reprogramming metastatic melanoma cells to assume a neural crest cell-like phenotype in an embryonic microenvironment. Proc Natl Acad Sci U S A. 2006;103(10):3752–7. PubMed Central PMCID: PMCPMC1450149. 10.1073/pnas.0506977103 16505384 PMC1450149

[9] Fonsato V, Collino F, Herrera MB, Cavallari C, Deregibus MC, Cisterna B, et al Human liver stem cell-derived microvesicles inhibit hepatoma growth in SCID mice by delivering antitumor microRNAs. Stem Cells. 2012;30(9):1985–98. PubMed Central PMCID: PMCPMC3468738. 10.1002/stem.1161 22736596 PMC3468738

[10] Bruno S, Collino F, Deregibus MC, Grange C, Tetta C, Camussi G. Microvesicles derived from human bone marrow mesenchymal stem cells inhibit tumor growth. Stem Cells Dev. 2013;22(5):758–71. 10.1089/scd.2012.0304 23034046

[11] Schatten G, Smith J, Navara C, Park JH, Pedersen R. Culture of human embryonic stem cells. Nat Methods. 2005;2(6):455–63. 10.1038/nmeth0605-455 16170868

[12] Xu RH, Peck RM, Li DS, Feng X, Ludwig T, Thomson JA. Basic FGF and suppression of BMP signaling sustain undifferentiated proliferation of human ES cells. Nat Methods. 2005;2(3):185–90. 10.1038/nmeth744 15782187

[13] Thomson JA, Itskovitz-Eldor J, Shapiro SS, Waknitz MA, Swiergiel JJ, Marshall VS, et al Embryonic stem cell lines derived from human blastocysts. Science. 1998;282(5391):1145–7. 9804556 10.1126/science.282.5391.1145

[14] Pera MF, Reubinoff B, Trounson A. Human embryonic stem cells. J Cell Sci. 2000;113 (Pt 1):5–10. 10591620 10.1242/jcs.113.1.5

[15] Postovit LM, Margaryan NV, Seftor EA, Kirschmann DA, Lipavsky A, Wheaton WW, et al Human embryonic stem cell microenvironment suppresses the tumorigenic phenotype of aggressive cancer cells. Proc Natl Acad Sci U S A. 2008;105(11):4329–34. PubMed Central PMCID: PMCPMC2393795. 10.1073/pnas.0800467105 18334633 PMC2393795

[16] Postovit LM, Seftor EA, Seftor RE, Hendrix MJ. A three-dimensional model to study the epigenetic effects induced by the microenvironment of human embryonic stem cells. Stem Cells. 2006;24(3):501–5. 10.1634/stemcells.2005-0459 16293574

[17] Khalkhali-Ellis Z, Galat V, Galat Y, Gilgur A, Seftor EA, Hendrix MJC. Lefty Glycoproteins in Human Embryonic Stem Cells: Extracellular Delivery Route and Posttranslational Modification in Differentiation. Stem Cells Dev. 9 2016; [Epub ahead of print]. 10.1089/scd.2016.0081 PMC5098129 27554431

[18] Valadi H, Ekstrom K, Bossios A, Sjostrand M, Lee JJ, Lotvall JO. Exosome-mediated transfer of mRNAs and microRNAs is a novel mechanism of genetic exchange between cells. Nat Cell Biol. 2007;9(6):654–9. 10.1038/ncb1596 17486113

[19] Abbott DE, Bailey CM, Postovit LM, Seftor EA, Margaryan N, Seftor RE, et al The epigenetic influence of tumor and embryonic microenvironments: how different are they? Cancer Microenviron. 2008;1(1):13–21. PubMed Central PMCID: PMCPMC2654360. 10.1007/s12307-008-0004-5 19308681 PMC2654360

[20] Giuffrida D, Rogers IM, Nagy A, Calogero AE, Brown TJ, Casper RF. Human embryonic stem cells secrete soluble factors that inhibit cancer cell growth. Cell Prolif. 2009;42(6):788–98. 10.1111/j.1365-2184.2009.00640.x 19732065 PMC6495992

[21] Lykke-Andersen S, Brodersen DE, Jensen TH. Origins and activities of the eukaryotic exosome. J Cell Sci. 2009;122(Pt 10):1487–94. 10.1242/jcs.047399 19420235

[22] Ratajczak J, Wysoczynski M, Hayek F, Janowska-Wieczorek A, Ratajczak MZ. Membrane-derived microvesicles: important and underappreciated mediators of cell-to-cell communication. Leukemia. 2006;20(9):1487–95. 10.1038/sj.leu.2404296 16791265

[23] Ratajczak J, Miekus K, Kucia M, Zhang J, Reca R, Dvorak P, et al Embryonic stem cell-derived microvesicles reprogram hematopoietic progenitors: evidence for horizontal transfer of mRNA and protein delivery. Leukemia. 2006;20(5):847–56. 10.1038/sj.leu.2404132 16453000

[24] Zhang S, Chu WC, Lai RC, Lim SK, Hui JH, Toh WS. Exosomes derived from human embryonic mesenchymal stem cells promote osteochondral regeneration. Osteoarthritis Cartilage. 2016. 10.1016/j.joca.2016.06.022 27390028

[25] Lai RC, Yeo RW, Padmanabhan J, Choo A, de Kleijn DP, Lim SK. Isolation and Characterization of Exosome from Human Embryonic Stem Cell-Derived C-Myc-Immortalized Mesenchymal Stem Cells. Methods Mol Biol. 2016;1416:477–94. 10.1007/978-1-4939-3584-0_29 27236691

[26] Khan M, Nickoloff E, Abramova T, Johnson J, Verma SK, Krishnamurthy P, et al Embryonic stem cell-derived exosomes promote endogenous repair mechanisms and enhance cardiac function following myocardial infarction. Circ Res. 2015;117(1):52–64. PubMed Central PMCID: PMCPMC4482130. 10.1161/CIRCRESAHA.117.305990 25904597 PMC4482130

[27] Raposo G, Stoorvogel W. Extracellular vesicles: exosomes, microvesicles, and friends. J Cell Biol. 2013;200(4):373–83. PubMed Central PMCID: PMCPMC3575529. 10.1083/jcb.201211138 23420871 PMC3575529

[28] Kastelowitz N, Yin H. Exosomes and microvesicles: identification and targeting by particle size and lipid chemical probes. Chembiochem. 2014;15(7):923–8. PubMed Central PMCID: PMCPMC4098878. 10.1002/cbic.201400043 24740901 PMC4098878

[29] Hosseini-Beheshti E, Pham S, Adomat H, Li N, Tomlinson Guns ES. Exosomes as biomarker enriched microvesicles: characterization of exosomal proteins derived from a panel of prostate cell lines with distinct AR phenotypes. Mol Cell Proteomics. 2012;11(10):863–85. PubMed Central PMCID: PMCPMC3494141. 10.1074/mcp.M111.014845 22723089 PMC3494141

[30] Malumbres M. Cyclin-dependent kinases. Genome Biol. 2014;15(6):122 PubMed Central PMCID: PMCPMC4097832. 10.1186/gb4184 25180339 PMC4097832

[31] Peurala E, Koivunen P, Haapasaari KM, Bloigu R, Jukkola-Vuorinen A. The prognostic significance and value of cyclin D1, CDK4 and p16 in human breast cancer. Breast Cancer Res. 2013;15(1):R5 PubMed Central PMCID: PMCPMC3672746. 10.1186/bcr3376 23336272 PMC3672746

[32] Hendzel MJ, Wei Y, Mancini MA, Van Hooser A, Ranalli T, Brinkley BR, et al Mitosis-specific phosphorylation of histone H3 initiates primarily within pericentromeric heterochromatin during G2 and spreads in an ordered fashion coincident with mitotic chromosome condensation. Chromosoma. 1997;106(6):348–60. 9362543 10.1007/s004120050256

[33] Van Hooser A, Goodrich DW, Allis CD, Brinkley BR, Mancini MA. Histone H3 phosphorylation is required for the initiation, but not maintenance, of mammalian chromosome condensation. J Cell Sci. 1998;111 (Pt 23):3497–506. 9811564 10.1242/jcs.111.23.3497

[34] Li X, Pei D, Zheng H. Transitions between epithelial and mesenchymal states during cell fate conversions. Protein Cell. 2014;5(8):580–91. PubMed Central PMCID: PMCPMC4130923. 10.1007/s13238-014-0064-x 24805308 PMC4130923

[35] De Toro J, Herschlik L, Waldner C, Mongini C. Emerging roles of exosomes in normal and pathological conditions: new insights for diagnosis and therapeutic applications. Front Immunol. 2015;6:203 PubMed Central PMCID: PMCPMC4418172. 10.3389/fimmu.2015.00203 25999947 PMC4418172

[36] Azmi AS, Bao B, Sarkar FH. Exosomes in cancer development, metastasis, and drug resistance: a comprehensive review. Cancer Metastasis Rev. 2013;32(3–4):623–42. PubMed Central PMCID: PMCPMC3843988. 10.1007/s10555-013-9441-9 23709120 PMC3843988

[37] Ciardiello C, Cavallini L, Spinelli C, Yang J, Reis-Sobreiro M, de Candia P, et al Focus on Extracellular Vesicles: New Frontiers of Cell-to-Cell Communication in Cancer. Int J Mol Sci. 2016;17(2):175 PubMed Central PMCID: PMCPMC4783909. 10.3390/ijms17020175 26861306 PMC4783909

[38] Desrochers LM, Bordeleau F, Reinhart-King CA, Cerione RA, Antonyak MA. Microvesicles provide a mechanism for intercellular communication by embryonic stem cells during embryo implantation. Nat Commun. 2016;7:11958 PubMed Central PMCID: PMCPMC4912619. 10.1038/ncomms11958 27302045 PMC4912619

[39] Medema JP. Cancer stem cells: the challenges ahead. Nat Cell Biol. 2013;15(4):338–44. 10.1038/ncb2717 23548926

[40] Niwa H, Miyazaki J, Smith AG. Quantitative expression of Oct-3/4 defines differentiation, dedifferentiation or self-renewal of ES cells. Nat Genet. 2000;24(4):372–6. 10.1038/74199 10742100

[41] Chambers I, Colby D, Robertson M, Nichols J, Lee S, Tweedie S, et al Functional expression cloning of Nanog, a pluripotency sustaining factor in embryonic stem cells. Cell. 2003;113(5):643–55. 12787505 10.1016/s0092-8674(03)00392-1

[42] Visvader JE, Lindeman GJ. Cancer stem cells: current status and evolving complexities. Cell Stem Cell. 2012;10(6):717–28. 10.1016/j.stem.2012.05.007 22704512

[43] Herreros-Villanueva M, Bujanda L, Billadeau DD, Zhang JS. Embryonic stem cell factors and pancreatic cancer. World J Gastroenterol. 2014;20(9):2247–54. PubMed Central PMCID: PMCPMC3942830. 10.3748/wjg.v20.i9.2247 24605024 PMC3942830

[44] Takahashi K, Yamanaka S. Induction of pluripotent stem cells from mouse embryonic and adult fibroblast cultures by defined factors. Cell. 2006;126(4):663–76. 10.1016/j.cell.2006.07.024 16904174

[45] Kumar SM, Liu S, Lu H, Zhang H, Zhang PJ, Gimotty PA, et al Acquired cancer stem cell phenotypes through Oct4-mediated dedifferentiation. Oncogene. 2012;31(47):4898–911. PubMed Central PMCID: PMCPMC3343184. 10.1038/onc.2011.656 22286766 PMC3343184

[46] Lin YL, Han ZB, Xiong FY, Tian LY, Wu XJ, Xue SW et al Malignant transformation of 293 cells induced by ectopic expressionof human Nanog. Mol Cell Biochem. 2011;351:109–116. Epub 2011 Jan 19 10.1007/s11010-011-0717-5 21246261

[47] Kim J, Zaret KS. Reprogramming of human cancer cells to pluripotency for models of cancer progression. EMBO J. 2015;34(6):739–47. PubMed Central PMCID: PMCPMC4369311. 10.15252/embj.201490736 25712212 PMC4369311

[48] Slack JM. Origin of stem cells in organogenesis. Science. 2008;322(5907):1498–501. 10.1126/science.1162782 19056975

[49] Ohlsson LB, Varas L, Kjellman C, Edvardsen K, Lindvall M. Mesenchymal progenitor cell-mediated inhibition of tumor growth in vivo and in vitro in gelatin matrix. Exp Mol Pathol. 2003;75(3):248–55. 14611816 10.1016/j.yexmp.2003.06.001

[50] Khakoo AY, Pati S, Anderson SA, Reid W, Elshal MF, Rovira, II, et al Human mesenchymal stem cells exert potent antitumorigenic effects in a model of Kaposi's sarcoma. J Exp Med. 2006;203(5):1235–47. PubMed Central PMCID: PMCPMC2121206. 10.1084/jem.20051921 16636132 PMC2121206

[51] Qiao L, Xu Z, Zhao T, Zhao Z, Shi M, Zhao RC, et al Suppression of tumorigenesis by human mesenchymal stem cells in a hepatoma model. Cell Res. 2008;18(4):500–7. 10.1038/cr.2008.40 18364678

