# Supplementary figures and images for: Reprogramming Malignant Cancer Cells toward a Benign Phenotype following Exposure to Human Embryonic Stem Cell Microenvironment
Source: PLoS One. 2017 Jan 9;12(1):e0169899. doi: 10.1371/journal.pone.0169899 (PMC5222525; doi:10.1371/journal.pone.0169899)

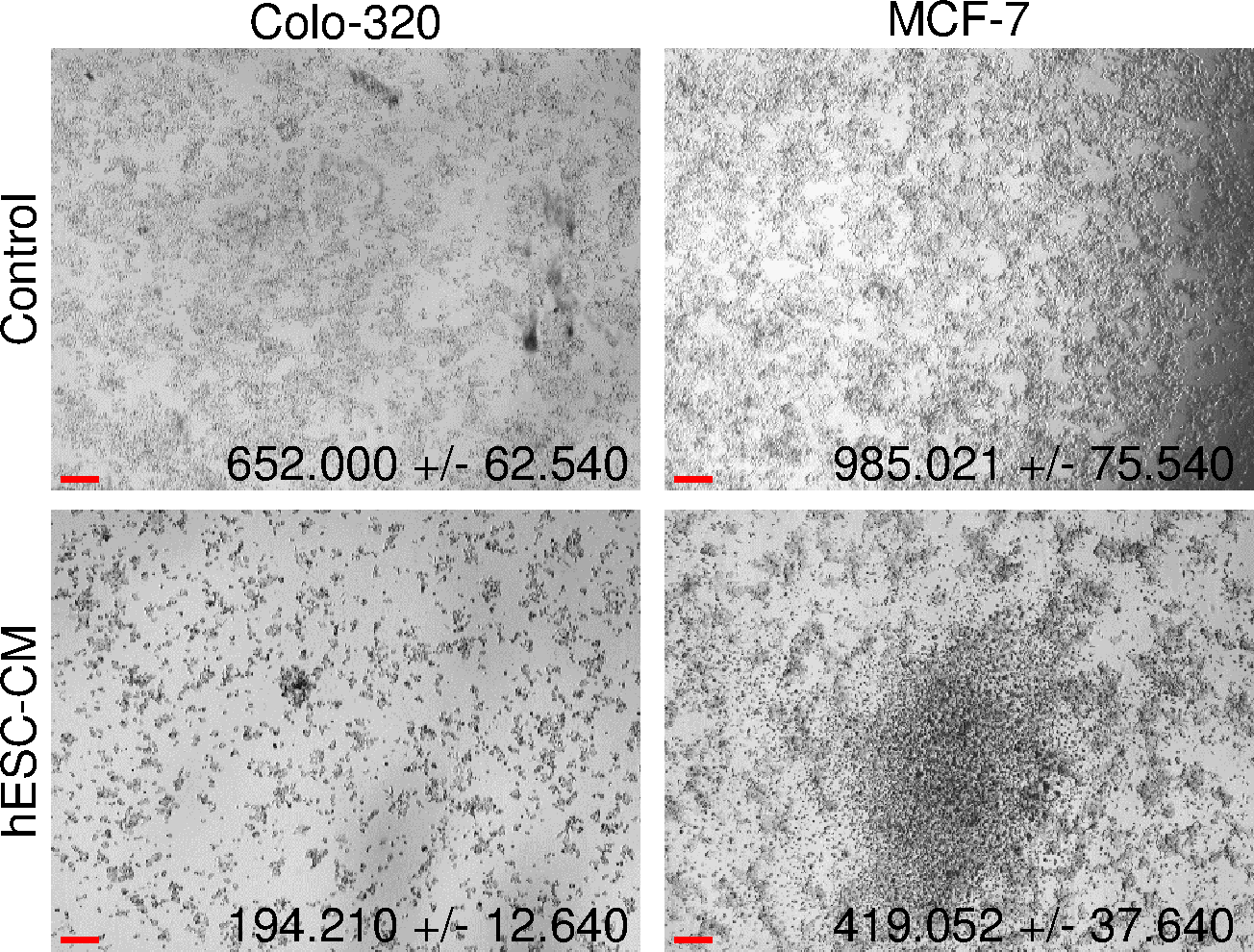

Supplement: S1 Fig — 100,000 Colo-320 and MCF-7 cancer cells were plated in control medium or hESC-CM for 3 days, and cells were analyzed for their growth potential. Bright field pictures of cell cultures at 3 days post-treatments. Note the significant reduction in cell density in cultures maintained in hESC-CM. Values are cells counts presented as mean +/- SD (n = 3 independent cultures, P < 0.05 when comparing control medium-treated cells to those treated with hESC-CM. Scale bar: 100 μm. (TIF) [file pone.0169899.s001.tif]

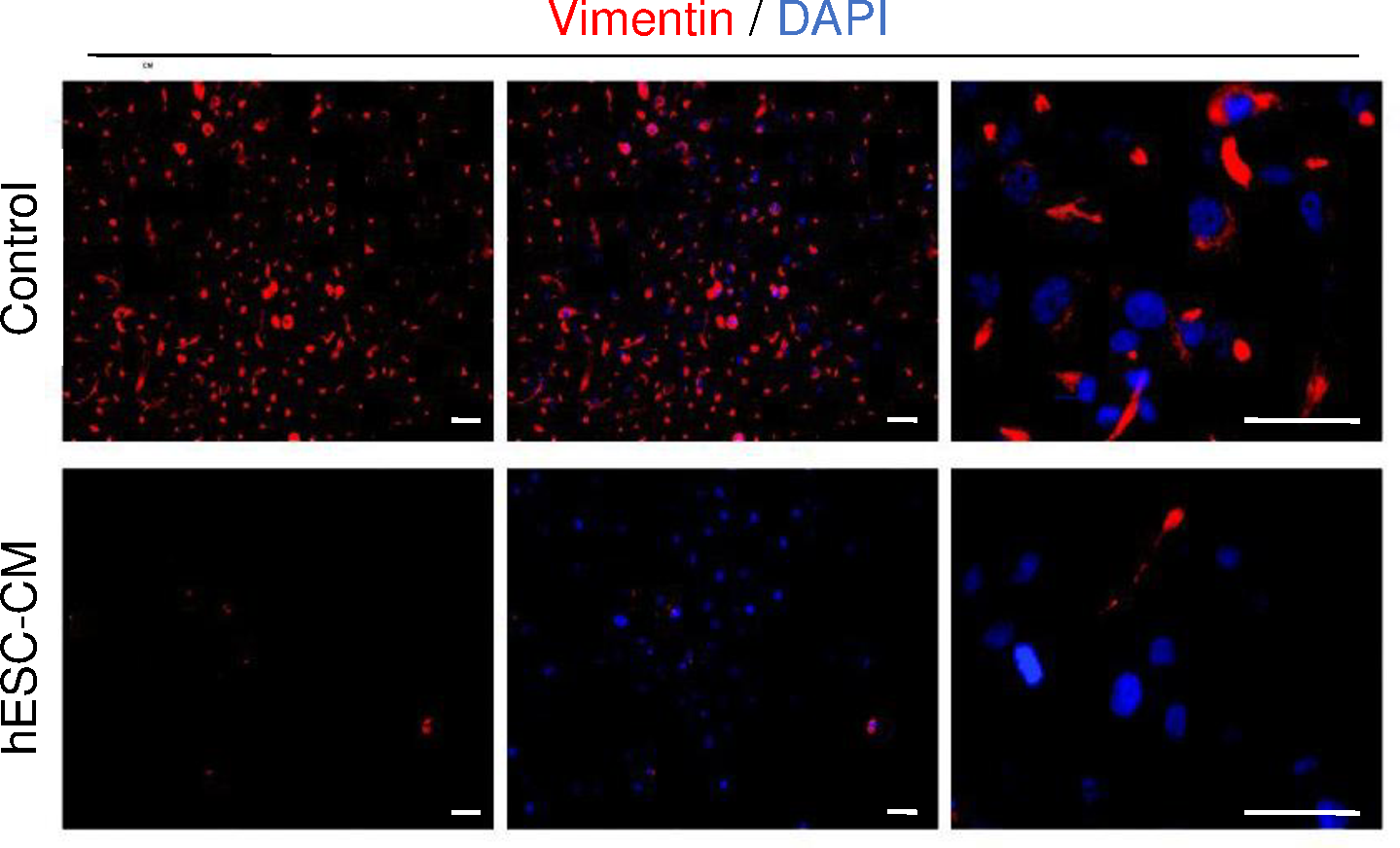

Supplement: S2 Fig — Cells were plated in control medium or hESC-CM for 3 days, and were analyzed by immunocytofluorometry for the expression of vimentin. Scale bar; 25 μm. (TIF) [file pone.0169899.s002.tif]

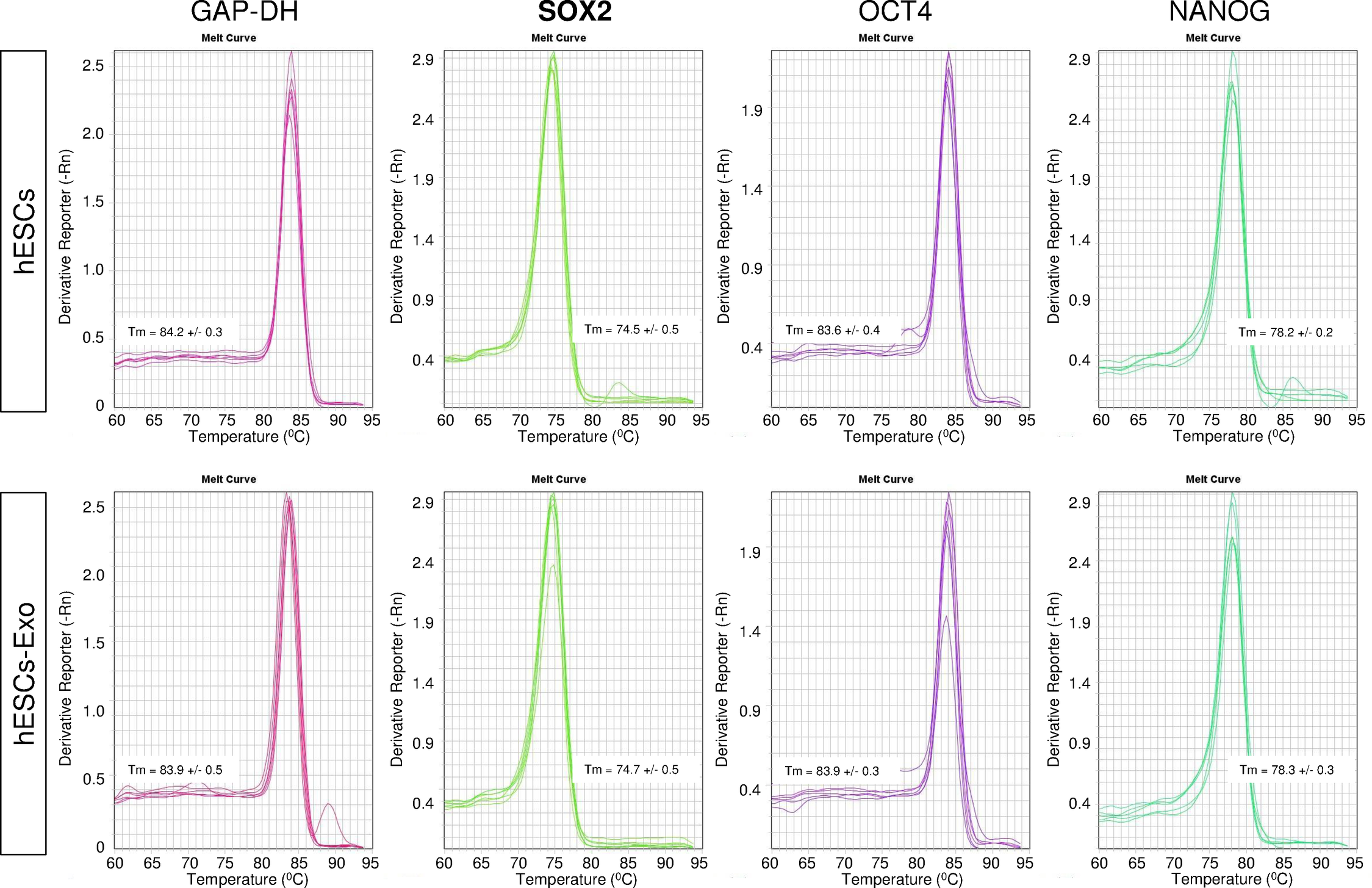

Supplement: S3 Fig — hESCs and hESCs-Exo isolated RNA were analyzed for the expression of pluripotency transcription factors SOX2, OCT4 and NANOG transcripts. Graphs display melt curves for the genes analyzed (n = 2 independent experiments repeated in triplicates). (TIF) [file pone.0169899.s003.tif]
